# Strychnine Poisoning in a Dog

**Published:** 1883-07

**Authors:** F. S. Billings


### CASE DEPARTMENT.

### I.—A CASE OF STRYCHNINE POISONING IN A DOG.

REPORTED BY F. S. BILLINGS, V. S.

A very valuable pointer belonging to a Mr. W. of Boston was found in convulsions in the yard early A. M. Saturday, May 5th. The usual neighborly assistance was volunteered, but without effect. At about four P. M. we were called in. Case looked serious. Patient much exhausted. Spasms very frequent. Tonic spasm of jaws, so that they could scarcely be opened. Eyes protruding. Respiration frequent and difficult. Visible mucous membranes cyanotic. Extremities cold. Nose dry. On the least touch patient went immediately into clonic spasms with opisthotonos of the most extreme degree. Eyes almost protruding from sockets and fixed. Treatment:

| | | |
|---|---|---|
| | Tortortus stibs, . . . | 0.72 |
| | Radix ipecac, . . , | 5.00 |
| M | Et div in pulv. no ii, . . | |

One to be given at once, second in half hour. Emesis did not follow the first. The second was followed by violent emesis, and great relaxtion of symptoms. Did not use anesthetics for this reason. The emetic was not given with any hope of relieving the stomach of poison, as too long a time had elapsed to render such a procedure justifiable, but to produce depression and relaxation. I think it is a mistake to torture such a patient with the application of the stomach pump in strychnine cases when so much time has elapsed. Such interference simply augments the spasms.

The next step was to introduce a solution of atropine subcutaneously. One gramme of a solution of 0.12 gramme to 30 aq. was used, and injections being made every hour. Aside from this, I ordered a mustard poultice to be applied over the spine for half an hour, and then to be washed off as easily as possible, after which patient was enclosed in blankets, heated warm as possible before the fire. After the emesis was thoroughly over, two compound cathartic pills U. S. were given, which operated during the night.

Visited patient again at 8 A. M. Sunday morning. All appearance of spasm had disappeared, and the do; was quite intelligent, but still very weak. Ordered beef tea and light diet, and left him as cured. Have heard nothing since.

